# The Relationship between Zinc Intake and Serum/Plasma Zinc Concentration in Children: A Systematic Review and Dose-Response Meta-Analysis 

**DOI:** 10.3390/nu4080841

**Published:** 2012-07-26

**Authors:** Victoria Hall Moran, Anna-Louise Stammers, Marisol Warthon Medina, Sujata Patel, Fiona Dykes, Olga W. Souverein, Carla Dullemeijer, Carmen Pérez-Rodrigo, Lluis Serra-Majem, Mariela Nissensohn, Nicola M. Lowe

**Affiliations:** 1 Maternal & Infant Nutrition & Nurture Unit, University of Central Lancashire, Preston PR1 2HE, UK; Email: FCDykes@uclan.ac.uk; 2 International Institute of Nutritional Sciences and Food Safety Studies, University of Central Lancashire, Preston PR1 2HE, UK; Email: ASkinner@uclan.ac.uk (A.-L.S.); MWarthon-medina1@uclan.ac.uk (M.W.M.); SPatel26@uclan.ac.uk (S.P.); NMLowe@uclan.ac.uk (N.M.L.); 3 Division of Human Nutrition, Wageningen University, PO Box 8129, 6700 EV Wageningen, The Netherlands; Email: Olga.Souverein@wur.nl (O.W.S.); Carla.Dullemeijer@wur.nl (C.D.); 4 Community Nutrition Unit, Bilbao City Council, Bilbao 48001, Spain; Email: bisaludpublica@wanadoo.es; 5 Department of Clinical Sciences, University of Las Palmas de Gran Canaria, Las Palmas de Gran Canaria 35016, Spain; Email: lserra@dcc.ulpgc.es (L.S.-M.); mnissensohn@acciones.ulpgc.es (M.N.)

**Keywords:** zinc, children, serum zinc, systematic review, dose-response, dietary recommendations, EURRECA

## Abstract

Recommendations for zinc intake during childhood vary widely across Europe. The EURRECA project attempts to consolidate the basis for the definition of micronutrient requirements, taking into account relationships among intake, status and health outcomes, in order to harmonise these recommendations. Data on zinc intake and biomarkers of zinc status reported in randomised controlled trials (RCTs) can provide estimates of dose-response relationships which may be used for underpinning zinc reference values. This systematic review included all RCTs of apparently healthy children aged 1–17 years published by February 2010 which provided data on zinc intake and biomarkers of zinc status. An intake-status regression coefficient (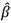) was calculated for each individual study and calculated the overall pooled 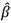 and SE (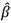) using random effects meta-analysis on a double log scale. The pooled dose-response relationship between zinc intake and zinc status indicated that a doubling of the zinc intake increased the serum/plasma zinc status by 9%. This evidence can be utilised, together with currently used balance studies and repletion/depletion studies, when setting zinc recommendations as a basis for nutrition policies.

## 1. Introduction

Suboptimal dietary zinc intake is increasingly recognised as an important public health issue. Although the lack of generally accepted biomarkers of zinc status has impeded estimation of the global prevalence of zinc deficiency, based on information regarding the amount of zinc present in national food supplies, it has been estimated that the risk of low dietary intake of absorbable zinc and consequent zinc deficiency affects between one-third and one-half of the world’s population [1] and rates of deficiency may approach 73% in some regions [2]. Although severe zinc deficiency is uncommon in European populations, marginal deficiency is likely to be much more prevalent [3], with associations to immune system dysfunction and restricted physical development [4]. Children are particularly vulnerable to suboptimal zinc status during periods of rapid growth that create increased zinc needs that may not be met [5,6]. It is estimated that over 450,000 deaths per year (4.4% of all mortalities) among children between six months and five years of age are attributable to zinc deficiency [7]. Zinc deficiency also has an impact of child morbidity, impairing growth and contributing to childhood stunting [8,9]. 

Physiological requirements for zinc peak at the time of the pubertal growth spurt, the onset of which varies according to gender. In girls the onset of the growth spurt (OGS) occurs at 10.1 years and peak height velocity (PHV) occurs at 12.0 years. In boys OGS and PHV occur at 11.8 and 14.2 years, respectively [10]. Even after the growth spurt has ceased, adolescents may require additional zinc to replete tissue zinc pools depleted during puberty [11]. Marginal zinc status during the pubertal growth spurt has been associated with slower skeletal growth, maturation, and reduced bone mineralisation [12,13,14]. As nearly a third of total skeletal mineral is accumulated in the 3–4 year period immediately after the onset of puberty [15] suboptimal zinc intake may have long-term consequences on bone health. 

Although a sensitive and specific biomarker has yet to be identified, a recent systematic review concluded that plasma (or serum) zinc concentration was responsive to both zinc supplementation and depletion and is the most widely used biomarker for zinc [16]. However, meta-analytic methods have not yet been used to model zinc status as a function of zinc intake levels. Understanding the relationship between dietary intake and micronutrient status is essential for deriving dietary recommendations. 

The recommendations for zinc intake during childhood vary widely across Europe and comparisons are difficult due to differences in categorisation. For example between 4 and 6 age categories are used to describe micronutrient recommendations in childhood with different age cut-off points being used by different European countries [17,18]. Recommendations for zinc intake differ between males and females at the age of 15 years in most countries, but also differ at the age of 10 years in some countries. Zinc intake values range from 2.9 to 10.0 mg/day in children aged 5 years, 5.7 to 15.5 mg/day (boys) and 4.6 to 15.0 mg/day (girls) in children aged 10–15 years [17]. The EURRECA project attempts to consolidate the basis for the definition of micronutrient requirements across Europe, taking into account relationships among intake, status and health outcomes, in order to harmonise these recommendations [19].

This paper presents a systematic review of the data from all available randomised controlled trials (RCTs) meeting EURRECA’s quality standard [20], which investigated zinc intake and biomarkers of zinc status, and combines these studies in meta-analyses to model zinc concentrations in serum or plasma as a function of zinc intake. 

## 2. Methods

### 2.1. Search Strategy

**Figure 1:**
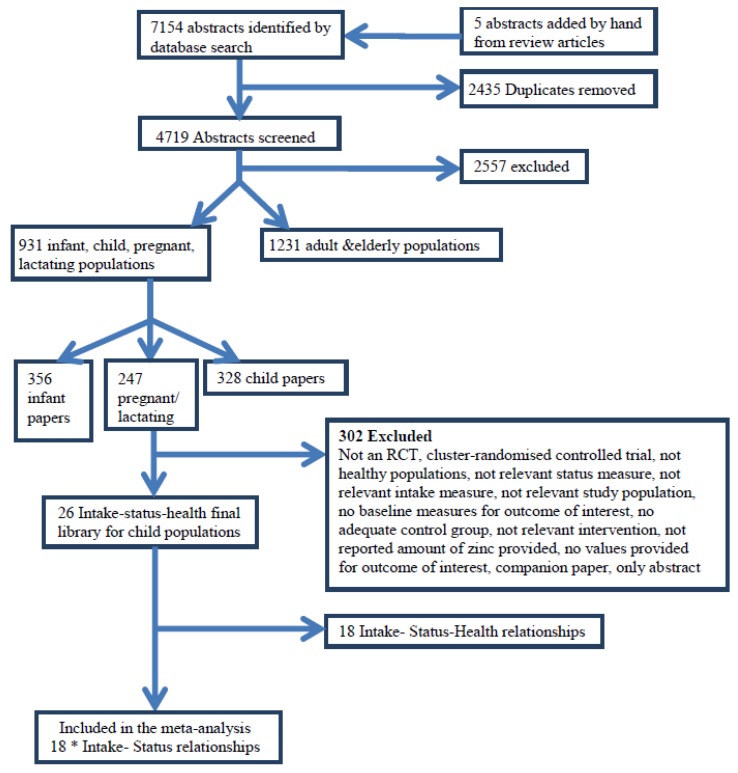
Study selection process for systematic review (* some papers reported more than one relationship).

This research was conducted within the framework of the European Micronutrient Recommendations Aligned (EURRECA) Network of Excellence that aims to identify the micronutrient requirements for optimal health in European populations (EURRECA [21]). This review was part of a wider review process to identify studies assessing the effect of zinc intake on different outcomes (biomarkers of zinc status and health outcomes). The wider searches were performed of literature published up to and including February 2010 using MEDLINE, Embase, and Cochrane using search terms for “study designs in humans” and “zinc” and “intake OR status”. Both indexing and text terms were used and languages included were restricted to those spoken in the EURRECA Network (English, Dutch, French, German, Hungarian, Italian, Norwegian, Polish, Spanish, Greek, and Serbian). The full Ovid MEDLINE search strategy can be found in Table 1. Reference lists of retrieved articles and published literature reviews were also checked for relevant studies. Authors were contacted to request missing data or clarify methods or results. The search process is illustrated in Figure 1.

**Table 1:** Search strategy: MEDLINE February 2010 [22].

No.	Search Term	Results
1	randomised controlled trial.pt.	280,821
2	controlled clinical trial.pt.	79,998
3	randomised.ab.	196,604
4	placebo.ab.	117,891
5	clinical trials as topic.sh.	146,242
6	randomly.ab.	145,491
7	trial.ab.	203,467
8	randomised.ab.	38,423
9	6 or 3 or 7 or 2 or 8 or 1 or 4 or 5	734,511
10	(animals not (human and animals)).sh.	4,482,479
11	9 not 10	642,665
12	(cohort* or “case control*” or cross-sectional* or “cross sectional” or case-control* or prospective or “systematic review*”).mp.	768,885
13	exp meta-analysis/ or expmulticenter study/ or follow-up studies/ or prospective studies/ or intervention studies/ or epidemiologic studies/ or case-control studies/ or exp cohort studies/ or longitudinal studies/ or cross-sectional studies/	1,013,635
14	13 or 12	1,203,767
15	14 not 10	1,154,385
16	11 or 15	1,599,094
17	((zinc or Zn or zinc sulphate or zinc gluconate or zinc acetate or methionine or zinc isotope*) adj3 (intake* or diet* or supplement* or deplet* or status or serum or plasma or leukocyte or concentration* or expos* or fortif* or urine or hair)).ti,ab.	16,681
18	Nutritional Support/ or Dietary Supplements/ or nutritional requirements/ or Breast feeding/ or exp infant food/ or bottle feeding/ or infant formula/	63,098
19	exp Nutritional Status/ or exp Deficiency Diseases/ or supplementation/ or diet supplementation/ or dietary intake/ or exp diet restriction/ or exp mineral intake/ or Diet/ or Food, Fortified/ or nutrition assessment/ or Nutritive Value/	176,014
20	(intake* or diet* or supplement* or deplet* or status or serum or plasma or leukocyte or concentration* or expos* or fortif* or urine or hair).ti,ab.	3,166,092
21	18 or 19 or 20	3,263,114
22	zinc/	41,027
23	22 and 21	20,745
24	23 or 17	26,943
25	24 and 16	2410

### 2.2. Criteria for the Consideration of Studies for This Review

Included studies were RCTs in apparently healthy child (human) populations aged from 1 to 17 years that supplied zinc supplementation either as capsules or part of a fortified meal. If supplemental zinc was provided as a component of a fortified meal, studies were only considered acceptable if zinc was the only constituent that was different between treatment groups. Only studies that measured zinc status as serum or plasma zinc were included; and those that reported sufficient data or had sufficient data obtainable from the authors to estimate 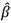 and SE (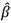) for the assumed linear relation on the log_e_–log_e_ scale. Studies were excluded if they were a group RCT (community trial), or were commentaries, reviews, or duplicate publications from the same study. Studies were excluded if children were hospitalised, had severe protein-energy malnutrition or a chronic disease or if supplemental zinc was provided for less than 6 weeks.

### 2.3. Selection of Articles

Of 4719 identified articles in the wider search on zinc intake, status and priority health outcomes in all populations, 2557 were excluded based upon screening of the title and abstract. Two independent reviewers screened 10% of the abstracts in duplicate and any discrepancies were discussed before screening the remaining references. Following subdivision into appropriate population groups the full texts of the 328 manuscripts were assessed to determine inclusion and exclusion by two independent reviewers and disagreements rectified through discussion. 302 studies were excluded because they did not meet the inclusion criteria. Of the remaining 26 studies, 8 studies were excluded as they had not investigated the relation between zinc intake and zinc related biomarkers, but related either intake or status directly to a health endpoint. For the purpose of this paper, 18 RCTs remained. Table 2 presents the characteristics of the included studies.

### 2.4. Data Extraction

For each of the identified manuscripts, data was extracted independently by two reviewers into a standardised database. Extracted data included population characteristics, dose of elemental zinc in intervention and placebo supplements, duration of the study, dietary intake of zinc, and mean concentration of zinc in plasma or serum at the end of the intervention period. Serum/plasma zinc concentrations were converted to µmol/L when applicable.

**Table 2:** Summary of included trials reporting the effect of dietary zinc intake on serum/plasma zinc status in children.

First Author, Year, Country	Participants	Treatment Groups (*n*)	Mean Zn Intake (mg/day)	Mean (SD) Plasma/Serum Zn (µmol/L)	Duration	Zinc Status Biomarker [Analytical Method]	Main Results
Mahloudji, 1975, Iran [23]	Males & females	Fe only (12);	5.65;	8.95 (1.80)	8 months	Plasma Zn [AAS]	No significant difference between plasma Zn of the supplemented and placebo groups
	aged 6–12 years	Fe + 20 mg/day Zn (13)	25.65	8.50 (1.93)			
Hambidge, 1979, USA [24]	Males & females	Male placebo (15);	6.3;	11.06 (2.23)	9 months	Plasma Zn [AES]	Plasma Zn significantly higher in Zn supplemented compared to placebo (girls and combined sexes only *p* < 0.05)
	aged 33–90 months	Male Zn FM 2.57 mg/day (20);	9.27;	11.85 (2.23)			
		Female placebo (14);	6.3;	10.61 (1.81)			
		Female Zn FM 2.57 mg/day (11)	9.27	11.96 (1.81)			
Walravens, 1983, USA [25]	Males & females	Placebo (16);	4.6;	11.32 (2.14)	12 months	Plasma Zn [AES]	No significant difference between plasma Zn of the supplemented and placebo groups
	aged 2–6 years	10 mg/day Zn (16)	15.9	10.86 (2.14)			
Gibson, 1989, Canada [26]	Males	Placebo (21);	6.4;	15.8 (3.5)	6 months	Serum Zn [AAS]	No significant correlation between serum Zn and dietary Zn levels
	aged 59–95 months	10 mg Zn/day (18)	16.7	17.9 (3.4)			
Cavan, 1993, Guatemala [27]	Males & females,	Placebo (74);	5.65;	14.9 (2.1)	25 weeks	Plasma Zn [AAS]	Plasma Zn significantly higher in Zn supplemented compared to placebo ( *p* < 0.01)
	mean age 81.5 (±7.0) months ^1^	10 mg Zn/day (71)	15.65	16.2 (2.9)			
Friis, 1997, Zimbabwe [28]	Males and females	Placebo (121);	5.65;	10.89 (2.5)	12 months	Serum Zn [AAS]	The decline in zinc concentration was significantly lower in the Zn supplemented group compared to the placebo group ( *p* < 0.02)
	aged 11–17 years	30–50 mg/day Zn (122)	45.65 ^2^	11.71 (2.4)			
Rosado, 1997, Mexico [29]	Males & females	Placebo (55);	5.65;	14.4 (4.45)	12 months	Plasma Zn [AAS]	Plasma Zn increased significantly in the Zn supplemented group over the 12 months period (*p* < 0.01)
	aged 18–36 months	20 mg Zn/day (54)	25.65	16.8 (5.88)			
Ruz, 1997, Chile [30]	Males & females	Placebo (33);	6.4;	17.7 (1.9)	6 months	Plasma Zn [AAS]	No significant difference between plasma Zn of the supplemented and placebo groups
	aged 27–50 months	10 mg/day Zn (36)	17.1	17.6 (2.2)			
Sandstead, 1998, China [31] (3 regions)	Males & females	*Chonqing* MN, no Zn (35);	5.65;	19.83 (4.12)	10 weeks	Plasma Zn [AAS]	Plasma Zn significantly higher in Zn supplemented compared to placebo (*p* < 0.05) in Chonqing and Quindgdao groups.
	aged 6–9 years	20 mg/day Zn + MN (35);	25.65;	23.6 (4.12)			
		*Quindgdao* MN, no Zn (36);	5.65;	20.42 (4.08)			
		20mg/day Zn + MN (36);	25.65;	22.97 (4.08)			
		*Shanghai* MN, no Zn (37);	5.65;	17.9 (2.75)			
		20 mg/day Zn + MN (37)	25.65	17.97 (2.75)			
Clark, 1999, UK [32]	Peripubertal females,	Placebo (19);	6.6;	12.6 (1.0)	6 weeks	Serum Zn [no method given]	Serum Zn significantly higher in Zn supplemented compared to placebo ( *p* < 0.001)
	mean age 12.2 (±0.3) years	15 mg Zn/day (23)	21.6	16.7 (4.9)			
Smith, 1999, Belize [33]	Males & females	Placebo (10);	5.65;	11.7 (0.68)	6 months	Serum Zn [AAS]	Serum Zn significantly higher in Zn supplemented compared to placebo (*p* < 0.001)
	aged 22–66 months	70 mg Zn/day (12)	75.65	13.5 (0.68)			
Munoz, 2000, Mexico [34]	Males & females	Placebo (54);	5.65;	14.3 (4.7)	6 months	Plasma Zn [AAS]	Serum Zn significantly higher in Zn supplemented compared to placebo (*p* < 0.0001)
	aged 18–36 months	20 mg/day Zn (47)	25.65	16.8 (5.6)			
Lopez de Romana, 2005, Peru [35]	Males & females	Fe FM (12);	4.71;	11.87 (1.88)	70 days	Plasma Zn [ICP-MS]	No significant differences in plasma Zn were found between treatments
	aged 3–4 years	Fe + 3 mg/day Zn FM (10);	8.72;	11.65 (1.25)			
		Fe + 9 mg/day Zn FM (12);	15.7	12.60 (1.51)			
Silva, 2006, Brazil [36]	Males & females aged 12–59 months ^3^	Placebo (30); 10 mg/day Zn (28)	5.65; 15.65	8.0 (0.58)13.4 (0.25)	4 months	Serum Zn [AAS]	Serum Zn significantly higher in Zn supplemented compared to placebo (*p* < 0.05)
Sandstead, 2008, USA (Mexican Americans) [37]	Males & females	MN, no Zn (25);	5.65;	15.4 (1.5)	10 weeks	Plasma Zn [AAS]	Mean plasma Zn increased significantly in both groups compared to baseline (*p* < 0.05)
	aged 6–7 years	20 mg/day Zn + MN (25)	25.65	15.6 (1.2)			
Wuehler, 2008, Ecuador [38]	Males & females	Placebo (56);	5.65;	10.6 (1.6)	6 months	Plasma Zn [ICP-MS]	The mean change in plasma zinc concentrations from baseline increased progressively with higher doses of supplemental Zn (*p* < 0.001)
	aged 12–30 months	3 mg Zn/day (50);	8.65;	12.3 (1.6)			
		7 mg Zn/day (52);	12.65;	13.3 (1.7)			
		10 mg Zn/day (54)	15.65	14.0 (1.7) ^4^			
de Oliveira, 2009, Brazil [39]	Pubescent males,	Placebo (26);	5.65;	16.9 (2.1)	12 weeks	Plasma Zn [ICP-MS]	Plasma Zn significantly higher in Zn supplemented compared to placebo (*p* < 0.05)
	mean age 13 (±0.4) years	22 mg Zn/day (21)	27.65	18.7 (3.5)			
Uckarde, 2009, Turkey [40]	Males & females	Placebo (109);	5.65;	19.19 (1.80)	10 weeks	Serum Zn [CS]	Both supplemented and placebo groups had significantly higher serum Zn at follow up (*p* < 0.05)
	aged 8–9 years	15 mg/day Zn (109)	20.65	19.50 (2.41)			

AAS, atomic absorption spectroscopy; AES, atomic emission spectroscopy; ICP-MS, inductively coupled plasma mass spectrometry; CS, caloric spectrophotometry; MN, micronutrients; FM, fortified meal; ^1^ all participants also received MN supplements; ^2^ children weighing <29.5 kg were given 30 mg Zn/day and those >29.5 kg were given 50 mg Zn/day, this figure is an average of the two doses; ^3^ all participants also received Fe fortified milk; ^4^ geometric means.

### 2.5. Data Synthesis

One study that included two zinc-treated groups and two control groups (males and females) was treated as two independent estimates in the analysis [24] and one study that included three zinc-treated groups and three control groups (from different regions of China) [31] and was treated as three independent estimates in the analysis. Where studies provided outcome data for two or more zinc-treated groups they were included as separate estimates in the meta-analysis [35,38]. In one study zinc status was measured at 6 months and 12 months in the same population [26] and only the measure at 6 months was used in the analysis (where *n* was the largest). If dietary intake of zinc (in addition to the intervention) was not reported we imputed a value of 5.65 mg/day, the mean dietary intake level of the RCTs (*n* = 7) that did report dietary zinc intake. As mean baseline serum/plasma zinc concentrations were infrequently reported, the serum/plasma zinc concentrations in the control group of the RCTs were used as a proxy of the baseline serum/plasma zinc concentrations for our analyses. 

### 2.6. Pre-Specified Potential Factors Modifying the Association

A pooled meta-analysis was performed combining the evidence from all the available RCTs. In addition, we investigated whether age, dose of zinc, duration of the supplementation, and type of supplement (zinc only *vs.* zinc with other micronutrients) were variables that modified the association. 

### 2.7. Statistical Analyses

As we wanted to estimate the dose-response relation between zinc intake and serum/plasma zinc, the data presented in the studies had to be transformed to a common statistic, namely a regression coefficient (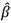) and the standard error (SE (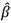)) of this regression coefficient. The transformations used to derive this common single-study estimate from the available summary statistics per study have been described elsewhere [41]. In short, we estimated an intake-status regression coefficient (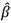) for each individual study, based on the assumption of a linear relation on the log_e_–log_e_-scale (natural logarithm of intake *vs.* natural logarithm of status). This shape of this linear relationship on the log_e_–log_e_-scale corresponds to a monotonic concave function on the original scale for β < 1. This shape is assumed to be realistic for the biological relationship between zinc intake and plasma/serum zinc concentrations. As the true dose-response curve is unknown, this approximation provides a practical methodology to estimate the dose-response relationship. We calculated the overall pooled 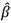 and SE (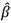) using random effects meta-analysis, which estimates the between-study variance using the method of DerSimonian and Laird and used this estimate to modify the weights used to calculate the summary estimate. Residual heterogeneity between studies was evaluated using the I^2^ statistic. Pre-specified potential factors that could modify the association were explored using stratified random effects meta-analyses. The statistical transformations to obtain 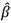
’s and SE (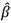)’s were performed using GenStat version 13-SP2 (VSN International Ltd. [42]) and the meta-analysis was performed using STATA version 11.0 (College Station, TX, USA), with statistical significance defined as *p* < 0.05.

### 2.8. Assessment of Risk of Bias in Included Studies

In order to assess the quality of the included studies and the risk of bias, indicators of internal validity were collected during data extraction (Table 2). Based on the indicators two independent reviewers assessed the overall risk of bias and disagreements resolved by discussion. The criteria for judging these indicators were adapted from the Cochrane Handbook for Systematic Reviews [43].

## 3. Results

Twenty-four estimates of zinc intake and serum/plasma zinc status in 18 RCTs with children were eligible for meta-analysis. All studies were RCTs published between 1975 and 2009 which reported zinc intake and plasma/serum zinc concentrations. The 24 estimates included 1722 participants in total with sample sizes ranging from 10 to 122. The median duration of the trials was 24 weeks (range 6–52 weeks). In 11 studies zinc was supplemented alone at doses ranging from 3 to 70 mg/day and in 7 studies participants also received other micronutrients. Zinc was provided with iron supplements [23] or iron fortified milk [36], as part of a fortified meal [24,35], and with other micronutrients [27,31,37]. The zinc dose ranged from 10 to 20 mg/day when combined with other vitamin/minerals and 2.57 to 9 mg/day when provided in fortified meals. Most studies (*n* = 7) provided the zinc supplements in the form of zinc sulphate, but others used zinc citrate [32], zinc gluconate [33], zinc carbonate [23], zinc methionine [29,34], amino acid chelate [27], and elemental zinc in a syrup [28,40]. Studies were conducted in Latin America (*n* = 9), North America (*n* = 4), Asia (*n* = 3), Africa (*n* = 1) and Europe (*n* = 1). Habitual zinc intakes ranged from 4.6 to 7.1 mg/day (where data was provided) and ages of children ranged from 2 to 17 years. Most studies included, but did not differentiate between, males and females, but one study provided separate male and female data [24], one included only females [32], and one only males [39].

The majority of studies (*n* = 13) reported that zinc supplementation significantly increased zinc plasma/serum status or significantly reduced the decline in zinc serum values compared to placebo. Of these, two studies also reported increased plasma/serum zinc concentrations in the placebo group. Five studies failed to find a significant relationship between zinc supplementation and zinc status [23,25,26,30,35], four of which provided zinc supplements or fortified meals with a zinc concentration of 10 mg/day or less.

**Figure 2:**
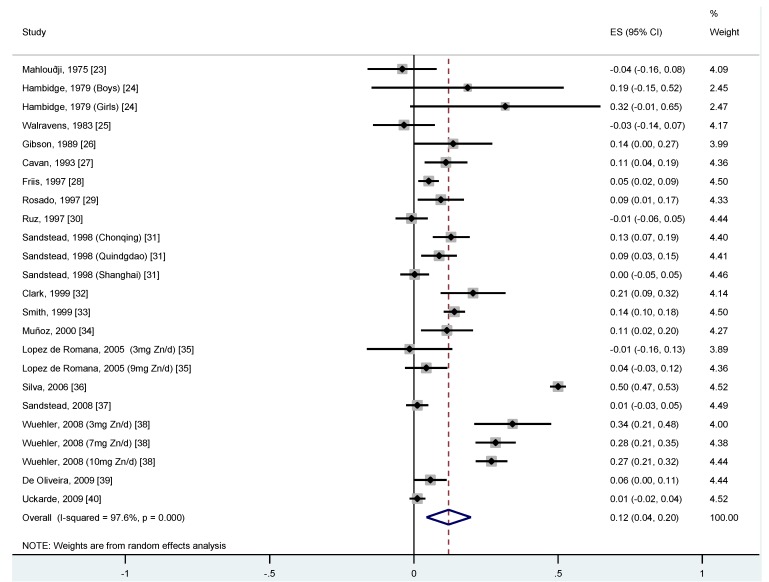
Random effects meta-analyses of RCTs evaluating the effect of dietary zinc on serum/plasma zinc in children. Beta’s represent the regression coefficients for the linear association between log_e_ transformed zinc intake and log_e_ transformed serum/plasma zinc status (lines represent the confidence intervals of each study).

Our meta-analysis of available studies suggested that zinc supplementation was associated with increased serum/plasma zinc concentrations. Combining the 18 RCTs in one meta-analysis yielded an overall pooled β-coefficient of 0.12 (95% CI 0.04, 0.20; *p* < 0.005; I^2^ 97.6%) (Figure 2). Since we applied a base-e logarithmic transformation on the zinc intake and serum/plasma zinc concentration before calculation of the study-specific 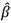 ’s, the overall 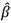 represents the difference in the log_e_ transformed predicted value of serum/plasma zinc status for each one-unit difference in the log_e_ transformed value in zinc intake. Therefore, an overall 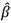 of 0.12 means that for every doubling in zinc intake, the difference in zinc serum or plasma concentration is 2^
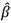
^ (2^0.12^ = 1.09), which is 9%. This means that a person with a zinc intake of 14 mg/day has a zinc serum/plasma concentration that is 9% higher than a person who has a zinc intake of 7 mg/day. 

As the physiological requirements for zinc peak at the time of the pubertal growth spurt, which generally occurs in girls between 10 and 15 years and in boys between 12 and 15 years, a separate subgroup analysis compared zinc intake and status according to age. As the onset of puberty was rarely assessed in papers, arbitrary age groups of <10 year, and ≥10 year were used. One study for which mean serum/plasma zinc values were given for children whose ages spanned both age groups were excluded from this analysis [23]. A meta-analysis of 18 studies performed in children under 10 years yielded an overall β of 0.13 (95% CI 0.04, 0.22; I^2^ 69.7%), compared to a meta-analysis of 3 studies performed in children over 10 years which yielded a β of 0.08 (95% CI 0.02, 0.15; I^2^ 97.9%), although care should be taken with interpreting this finding as the latter analysis is based on limited available data.

There is statistical evidence for substantial between-study heterogeneity in the overall meta-analysis (I^2^ 97.6%, *p* < 0.0001). To evaluate potential sources of heterogeneity, the variables duration, age, dose of zinc, and zinc status of the placebo groups (as a proxy for baseline zinc status) were added simultaneously to a meta-regression model as continuous variables. The analysis revealed that only zinc status of the placebo group was a statistically significant determinant of the overall β. The model explained 26.5% of between-study variance but the residual variation due to heterogeneity remained high (91.7%). It is important to note that serum/plasma zinc levels of the placebo groups were used as a proxy of baseline serum/plasma zinc as the mean baseline serum/plasma zinc levels were infrequently reported in the papers, however it does suggest that the β vary according to zinc status.

Table 3 summarises the internal validity of the included studies, assessed as described in the methods section. The risk of bias was high in all but three where the risk was low/moderate [25,37,38]. Papers were given a high risk of bias rating due to insufficient information provided on sequence generation and/or allocation, drop-outs and funding bodies.

**Table 3:** Assessment of validity of included RCTs reporting zinc intake and serum/plasma zinc in children (adapted from the Cochrane Handbook [43]).

Author, Year	Adequate Sequence Generation	Allocation Concealment Adequate	Blinding Adequate	Dropouts Adequate and Outcome Data Complete	Funder Adequate	Lack of other Potential Threats to Validity	Overall Risk of Bias
Mahloudji, 1975 [23]	Unclear	Yes	Unclear	Unclear	Yes	Unclear	High
Hambidge, 1979 [24]	Unclear	Unclear	Yes	Unclear	No	Unclear	High
Walravens, 1983 [25]	Unclear	Yes	Yes	Yes	Yes	Yes	Moderate
Gibson, 1989 [26]	Unclear	Yes	Yes	Yes	No	Yes	High
Cavan, 1993 [27]	Unclear	Yes	Yes	Unclear	Yes	No	High
Friis, 1997 [28]	Unclear	Yes	Yes	Yes	Yes	Yes	High
Rosado, 1997 [29]	Unclear	Yes	Yes	Unclear	Yes	Yes	High
Ruz, 1997 [30]	Unclear	Yes	Yes	Unclear	Yes	Yes	High
Sandstead, 1998 [31]	Unclear	Unclear	Yes	Unclear	No	No	High
Clark, 1999 [32]	Yes	Yes	Yes	Unclear	No	Unclear	High
Smith, 1999 [33]	Unclear	Unclear	Unclear	Yes	Yes	Yes	High
Muñoz, 2000 [34]	Unclear	No	Yes	Yes	Nor	Yes	High
Lopez de Romana, 2005 [35]	Unclear	Unclear	Unclear	Yes	Yes	Yes	High
Silva, 2006 [36]	Unclear	Unclear	No	Yes	No	Yes	High
Sandstead, 2008 [37]	Yes	Yes	Yes	Unclear	No	Yes	Moderate
Wuehler, 2008 [38]	Yes	Unclear	Yes	Yes	Yes	Yes	Low
de Oliveira, 2009 [39]	Unclear	Unclear	No	Unclear	No	Yes	High
Uckarde, 2009 [40]	Unclear	Yes	Yes	Yes	No	Yes	High

## 4. Discussion

This study is unique in providing an estimate of the dose-response relationship of zinc intake and serum/plasma zinc concentrations in children aged 1–17 years. Similar to findings published in an earlier systematic review [44], this meta-analysis of 24 estimates in 18 RCTs found a significant effect of zinc intake and serum/plasma zinc concentrations in children. In addition we have provided an estimate of the dose-response relationship between zinc intake and serum/plasma concentrations. An overall 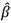 of 0.12 means that for every doubling in zinc intake, the difference in zinc serum or plasma concentration is 9%. In other words, a child with a zinc intake of 14 mg/day has a zinc serum/plasma concentration that is 9% higher than a person who has a zinc intake of 7 mg/day. It is important to note however that, due to homeostatic regulatory mechanisms, the amount of dietary zinc absorbed decreases as intake increases, and plasma zinc concentration is homeostatically controlled within a narrow physiological range, therefore this dose response relationship can only be applied to the range of intakes used to derive this relationship. The studies included in this meta-analysis were different in a number of aspects, such as using various designs, follow-up times, zinc doses, and populations. Therefore, it is no surprise that, when combining these studies in a meta-analysis, a large heterogeneity is observed between the studies (I^2^ = 97.6% *p* = 0.0001). This between-study heterogeneity may be caused by methodological factors, such as biological factors, e.g., differences in study population characteristics (age, socio-economic status), differences in doses of provided zinc (amount, one or more doses per day, study duration). We have considered the dose of zinc provided, study duration, age, and supplement type and these factors did not significantly explain the between-study heterogeneity. An individual participant data meta-analysis may have provided a more conclusive explanation of the between-study heterogeneity in this meta-analysis. However, this type of analysis would involve the input of raw individual participant data provided by the original study investigators for re-analysis and combination in a pooled analysis and as such would be a major undertaking in terms of time, costs, and collaboration. Moreover, an inability to include individual participant data from all relevant studies could introduce selection bias. The meta-analytic approach used in this paper is not an attempt to accurately describe the biological relation between actual zinc intake and zinc concentrations in blood under strict experimental conditions and on an individual level, but rather to simulate a dose-response relationship between zinc intake and status that is useful for surveillance studies with a public health point of view and, as such, deliberately incorporates the differences between dietary assessment methods, laboratory assessment methods and participant characteristics to ensure a broad external validity. Thus, the heterogeneity reflects the lack of standardisation of methods and the true heterogeneity between study populations and necessarily enters as uncertainty into the application of such data for public health purposes [45].

The relationship observed between serum/plasma zinc concentration and zinc intake may have been weakened by the limitation of this particular biomarker for zinc status. It is well established that plasma zinc concentration can fall in response to factors unrelated to zinc status or dietary zinc intake, such as infection, inflammation, exercise, stress or trauma. Conversely, tissue catabolism during starvation can release zinc into the circulation, causing a transient increase in circulating zinc levels. Postprandial plasma zinc concentrations have been reported to fall up to 19% [46]. Twelve studies used fasting blood samples in their analyses (usually overnight). Other factors related to the adequacy of serum/plasma sampling, such as storage and separation of samples, was often inadequately reported. Whilst all studies included in the analysis were undertaken in individuals without chronic disease or severe protein-energy malnutrition, other factors such as stress, infection and inflammation may also have gone unreported. For example, only three studies screened for parasitic infection [28,30,36]. Clearly such confounders have a strong influence on the interpretation of plasma zinc concentrations. However, as more sensitive indices of zinc status have yet to be identified, plasma serum zinc remains by far the most commonly used biomarker of zinc status [16]. 

It is important to note that the majority of studies in our meta-analysis (*n* = 13 of 18) were conducted in countries where participants are likely to have dietary patterns with low-moderate zinc bioavailability with higher fibre and phytate content which may have weakened the overall β. Although suboptimal zinc status may be caused by inadequate dietary intake of zinc in some cases, inhibitors of zinc absorption are likely the most common causative factor [47], and recent evidence in adults suggests that the inhibitory effect of dietary factors such as phytate on zinc absorption is likely to be much greater than previously recognised [48], although whether this is the case for children is less certain [49]. Indeed, a proxy measure for initial nutritional status of participants (zinc concentration in placebo groups) was found to be a significant effect modifier of β. However as very few studies reported baseline zinc or gave details of the concentration of indigestible zinc binding ligands in participants’ diets we were unable to investigate this important effect further.

Zinc was given in combination with other micronutrients including iron in several studies. As iron and zinc are known to compete for absorption [50] it is possible that iron supplements may impair child zinc status [47]. It is possible therefore that additional supplementation of iron may have reduced the effect of zinc supplementation on zinc plasma levels. A review on the interaction between zinc and iron in supplementation trials reported that, in the 4 RCTs reviewed, addition of iron to zinc supplementation did not affect plasma zinc status in children [51]. As three of the included trials were in infants aged 4–6 months and as the iron-regulatory mechanisms of infants may differ before and after 9 months of age [52], further studies in older age groups are needed to understand more fully the interaction effects of micronutrient supplementation.

To conduct our meta-analysis some assumptions related to the availability of the required data or related to statistical issues had to be made. The meta-analysis required transformations of the intake and biomarker data to a common scale, as the studies included in our meta-analyses had different ways of reporting the relation between zinc and biomarkers of zinc status in blood. We standardised the different ways of reporting by transformation of both the intake and biomarker data to double log_e_-scale, which allowed us to derive a standardised estimate from each study of the regression coefficient and its standard error as a basis for comparing these heterogeneously reported results. We also assumed a linear relationship on the double log_e_-scale. This rigorous but flexible transformation allowed us to pool β’s and report these as a dose-response relationship between zinc intake and serum/plasma zinc concentrations. As compared to a conventional meta-analysis of mean differences between high and low exposed subjects, a linear relationship on the double log_e_-scale with a slope lower than 1 allows us to model biomarker levels as a non-linear but monotonic concave function of dose, which is considered a more likely shape in biology. The meta-analyses were conducted within the context of the EURRECA project as a means to provide additional evidence for underpinning reference values for zinc intake of populations [53]. Whether the dose-response relationship, as provided in this paper, could be used as either qualitative or quantitative evidence to substantiate the daily zinc intake dose necessary to achieve normal or optimal levels of biomarkers for zinc status, remains a matter of discussion regarding the cut-off levels for biomarkers of zinc and the predictive value of serum/plasma zinc concentration for relevant functional health outcomes such as growth, immune function, cognitive function and psychomotor development.

Due to the wide heterogeneity that exists in the published literature on the relation between zinc intake and zinc status, such data cannot be combined in a conventional meta-analysis. Our paper not only provides a useful summary of this data in a systematic review, but also demonstrates a new meta-analytic approach to summarise all this data while appreciating the heterogeneity of it. The mathematical basis of this novel approach has recently been published [41] and is beyond the scope of the paper under review here, but in summary, we modelled a dose-response relation as a monotonic concave function between zinc intake and biomarkers. This is an innovative way to use all the data available, albeit heterogeneous data, to model the dose-response relationship; information which is essential when appraising micronutrient recommendations. When more research on zinc intake and status becomes available, this meta-analytical approach can be improved to strengthen the evidence on which we base our zinc recommendations. 

## 5. Conclusion

In conclusion, based on 24 estimates among 1722 participants, the results indicate that a doubling of zinc increases plasma/serum levels by 9%. Although it is recognised that serum/plasma zinc is a somewhat flawed biomarker of zinc status, in the absence of suitable alternatives it remains by far the most commonly used method and as such this review provides a valuable source of information for those seeking to assess zinc nutriture. As serum/plasma zinc levels are considered intermediates in the causal path to health benefits and because the heterogeneity between study results reflects real uncertainty in the evidence base, these issues must be taken into account when our dose-response data are used as complementary evidence for underpinning zinc reference values.
